# Prediction of Cancer Prevention: From Mammogram Screening to Identification of *BRCA1*/*2* Mutation Carriers in Underserved Populations

**DOI:** 10.1016/j.ebiom.2015.10.022

**Published:** 2015-10-21

**Authors:** Linda S. Robinson, Ashley Hendrix, Xian-Jin Xie, Jingsheng Yan, Sara Pirzadeh-Miller, Mary Pritzlaff, Parker Read, Sarah Pass, David Euhus, Theodora S. Ross

**Affiliations:** a Department of Cancer Genetics, University of Texas Southwestern Medical Center's Harold Simmons Comprehensive Cancer Center, Dallas and Moncrief Cancer Institute, Fort Worth, TX, USA; b Department of Biostatistics and Clinical Sciences, University of Texas Southwestern Medical Center, Dallas, USA; c Harold Simmons Comprehensive Cancer Center, University of Texas Southwestern Medical Center,Dallas, TX, USA; d Department of Surgery, Johns Hopkins Hospital, Baltimore, MD, USA; e Department of Internal Medicine, University of Texas Southwestern Medical Center, Dallas, TX, USA

**Keywords:** Hereditary breast and ovarian cancer syndrome, Population screening, BRCA1/2, Genetic testing, Underserved

## Abstract

**Background:**

The US Preventative Service Task Force recommends that physicians perform a genetic risk assessment to identify women at risk for *BRCA1*/*2* mutations associated with hereditary breast and ovarian cancer (HBOC) syndrome. However, outcomes data after a diagnosis of HBOC syndrome especially in diverse populations, are minimal. Here we asked if genetic screening of high-risk underserved women identified in the mammogram population reduces cancer incidence.

**Methods:**

We evaluated 61,924 underserved women at screening mammography for family histories suggestive of HBOC syndrome over the course of 21 months. Data were collected retrospectively from patients at two safety net hospitals through chart review. A computer model was used to calculate the long-term effect of this screening on cancer incidence by assessing both the mutation detection rate and the completion of prophylactic surgeries in *BRCA1*/*2* mutation carriers.

**Findings:**

We identified 20 of the 85 (23.5%) expected *BRCA1*/*2* mutation carriers in the underserved population. The frequencies of prophylactic mastectomies and oophorectomies in the mutation carriers were 25% and 40%, respectively. Using these data, our model predicted only an 8.8% reduction in both breast and ovarian cancer in the underserved patients. This contrasts with a 57% reduction in breast cancer and 51% reduction in ovarian cancer in an insured reference population. Our data indicate that underserved patients with HBOC syndrome are difficult to identify and when identified are limited in their ability to adhere to NCCN guidelines for cancer prevention.

**Interpretation:**

Screening for women at risk for HBOC syndrome in mammogram populations will only prevent cancers if we can increase compliance with management guidelines. This study provides prototypic baseline data for step-wise analysis of the efficacy of the use of family history analysis in the mammography setting for detection and management of HBOC syndrome.

## Highlights

• We report outcomes from screening underserved populations at mammography for risk of having a hereditary cancer syndrome.
• Despite the screening program, discovery of *BRCA* mutations and prophylactic surgery rates were low in the underserved.
• A prototype model used the study's outcomes to predict population cancer reduction and identify how to improve prevention.

This study evaluated 96,055 individuals at mammography for a family history of cancer to identify BRCA mutation carriers who have a genetic predisposition to breast and ovarian cancer.

The level of success in identification of high risk individuals and completion of cancer prevention activities was used to quantify cancer prevention as a result of our screening program.

We observed suboptimal outcomes, especially in the underserved populations, where screening for high risk individuals did not reduce cancer incidence. This study's model can be used for screening programs to find high risk patients and identify ways to improve outcomes for these patients.

## 1 Introduction

It is unknown what impact screening underserved populations for genetic risks for cancer has both on patients' compliance with management guidelines and on the ultimate goal of reducing cancer incidence. The hereditary breast and ovarian cancer (HBOC) syndrome is among the most common inherited cancer predisposition syndromes (Ford, D, et al., 1998, Lindor, NM, et al., 2008), making groups of patients with *BRCA1*/*2* mutations suitable populations to use to examine the benefits of screening. For patients carrying these mutations, approximately 57% of women with a *BRCA1* mutation and 49% of women with a *BRCA2* mutation will develop breast cancer by the age of 70 (Chen et al., 2006). The average risk for ovarian cancer in patients with a *BRCA1* mutation is 40% and in patients with a *BRCA2* mutation, 18% (Chen et al., 2006). While these are rare syndromes that cause an estimated 5–10% of all breast and 23% of ovarian cancers, individuals carrying these mutations have the highest cancer incidence of any known group. It has been estimated that 348,274 women in the United States over the age of 20 have a *BRCA* gene mutation (Drohan et al., 2012). After 14 years of *BRCA* gene testing, we have only identified approximately 50,000 of these female mutation carriers (Drohan et al., 2012). Seventy percent of *BRCA* mutation carriers with breast or ovarian cancer and 95% of unaffected *BRCA* mutation carriers remain unidentified (Drohan, B, et al., 2012, Bellcross, CA, et al., 2009).

Population based screening programs to identify more HBOC patients in the primary care setting have been proposed by many. The US Center for Disease Control's (CDC's) office of Public Health Genomics has classified screening for a family history of cancer to identify patients with HBOC syndrome as a tier one application (i.e. evidence supports their implementation into medical practice and surveillance) (Khoury, MJ, et al., 2010, Dotson, WD, et al., 2014). However, population screening outcomes data, especially in underserved communities, are scant. In 2013, the US Preventative Services Task Force (USPSTF) recommended genetic risk assessment and, if warranted, *BRCA* mutation testing in asymptomatic women with a family history of breast and ovarian cancer (Nelson et al., 2014). The goal of this recommendation is to both minimize unproductive genetic referrals and identify more *BRCA1*/*2* mutation carriers. A number of brief family history of cancer screening tools have been successfully used to identify HBOC patients in small populations and are recommended by the USPSTF (Bellcross, CA, et al., 2009, Dominguez, FJ, et al., 2005, Evans, DG, et al., 2004, Gilpin, CA, et al., 2000, Hoskins, KF, et al., 2006). However, there are no studies that quantitate the reduction of cancer cases as a result of large-scale population screening to identify patients with HBOC syndrome.

The goal of population screening for at-risk patients should be not only to identify mutation carriers, but also to improve their compliance with management recommendations to prevent cancers. However, since compliance rates are unknown, so too are the benefits of screening, especially in underserved communities. Here we measured patient compliance to NCCN management guidelines and rates of prophylactic surgery in an underserved HBOC syndrome patient cohort that was identified based on their family history of cancer at the time of a screening mammogram. We calculated the change in population cancer incidence over time based on the number of *BRCA1*/*2* mutation carriers we identified and the rate of prophylactic surgeries done to identify opportunities to improve cancer prevention.

## 2 Patients and Methods

### 2.1 Participants

From October 1st, 2011 to June 30th, 2013 (21 months), we screened for a family history of cancer in 96,055 mammogram patients for possible HBOC syndrome (Bellcross, CA, et al., 2009, Bellcross, C., 2010). The underserved study population was from two county hospitals (n = 61,924; Parkland Memorial and John Peter Smith (JPS) hospitals in Dallas and Fort Worth, Texas, respectively) and for reference a primarily insured population from our private hospital (n = 34,131; UT Southwestern Medical Center in Dallas). Parkland and JPS hospitals are the only safety net facilities in their counties serving patients with incomes below 200% of the federal poverty level. Many of the physicians at Parkland Memorial also work at UT Southwestern Medical Center. However, the staff at the JPS hospital is composed of private community board certified physicians. The genetic counselors at all sites are from the UT Southwestern cancer genetics program. Both safety net facilities have had a cancer genetics program in place since 2008 and their physicians are experienced in the management of hereditary cancer syndromes (Pirzadeh, 2009). All facilities offer HBOC patients prophylactic surgeries.

The 96,055 patients represented all women attending mammography during this time. Of those, 4% (n = 3928 patients) did not provide family history information. Because of this they were considered non-informative and were not studied further. Our study populations spanned 6 counties covering an area of 4688 mi^2^, including both rural and urban patients. The CancerGene Connect software was used to track all the patients identified as high-risk and their compliance at each step of the screening process (Pritzlaff et al., 2014). All participants provided informed consent. This study was approved by the Institutional Review Board at UT Southwestern Medical Center.

### 2.2 HBOC Screening

**Fig. 1:**
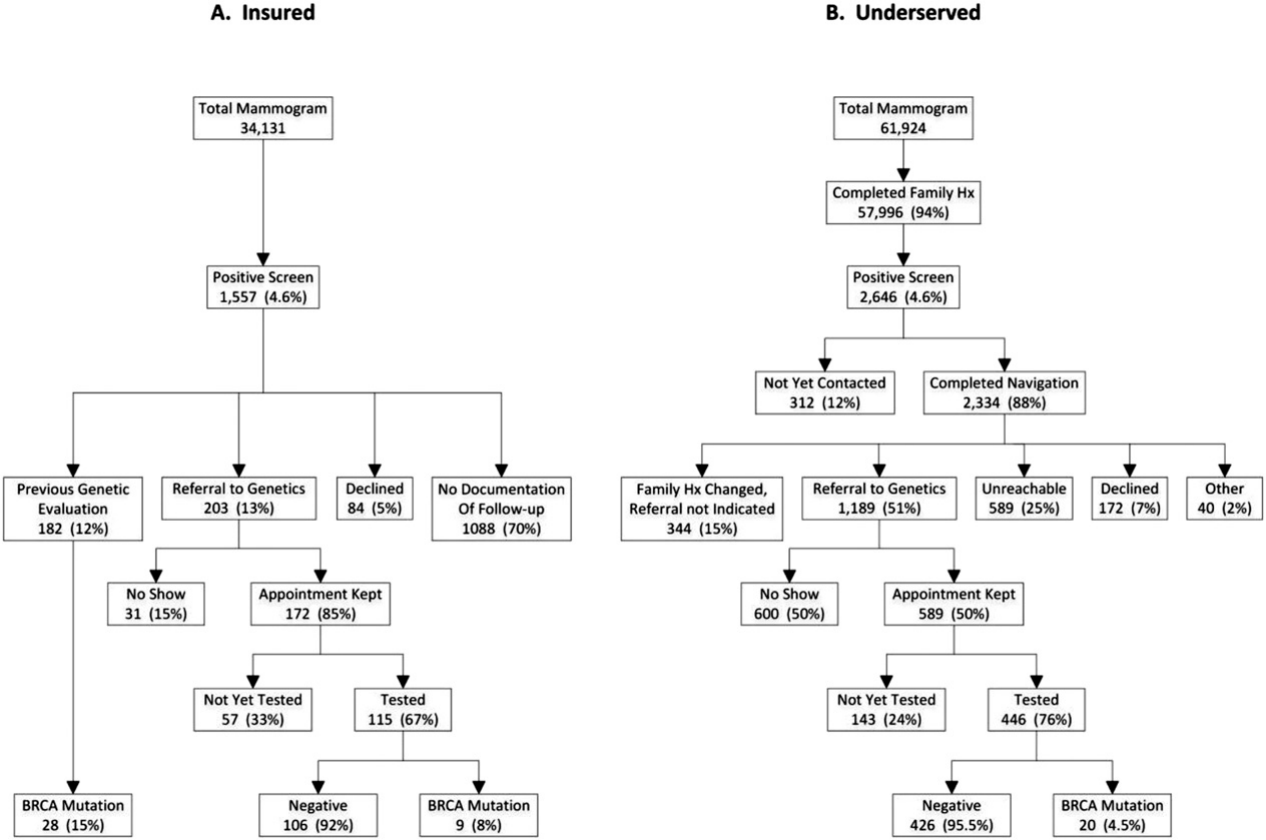
Mutation detection rates from the mammogram screening program. Mammogram patients who completed the RST questionnaire and were positive for family history suggestive of HBOC were then ushered through to genetics for further evaluation. A) Insured population and B) underserved population. This population completed navigation, defined as contacting the patient at least twice for a genetic referral.

To implement family history of cancer screening for possible HBOC syndrome in both the insured and underserved communities, we used the referral screening tool (RST) developed by the CDC (Bellcross, CA, et al., 2009, Bellcross, C., 2010). This tool identified high-risk patients based on a family history of cancer who were appropriate for HBOC testing in both populations. We modified the RST to include breast cancer patients diagnosed before the age of 45 and all ovarian cancer patients. This was to ensure that we could identify high-risk patients based on the NCCN genetic testing guidelines, including those who may not have previously been offered genetic services. As expected, approximately 5% of mammogram patients warranted a genetics referral (Fig. 1) (Bellcross, CA, et al., 2009, Dominguez, FJ, et al., 2005, Brannon Traxler, L, et al., 2014). A genetic patient navigator called all underserved patients identified as high-risk and offered them follow-up services. This was not done for the private hospital population, as a navigator was not funded. Information about the family history and recommendations for a genetics referral were incorporated into the mammogram screening report sent to the ordering physician. High-risk patients from both populations who attended a genetic counseling appointment were offered a *BRCA1*/*2* test if warranted (based on NCCN guidelines). Genetic testing was performed by either Myriad Genetic Laboratories or Ambry Genetics. The testing included *BRCA1*/*BRCA2* sequencing and duplication and deletion analysis. Medical records were retrospectively reviewed to determine the number of mutation carriers detected and if mutation carriers followed NCCN management guidelines.

### 2.3 Expected HBOC Mutation Frequency

To measure the impact of the RST screen for HBOC syndrome, quantitation of the mutation carrier detection rate compared to the expected rate is needed, as well as evaluation of prophylactic surgery rates. The estimated frequency in the general (non-Ashkenazi Jewish) population of *BRCA1* mutations is 0.058% and of *BRCA2* mutations, 0.068% (Antoniou et al., 2002). Based on the US Census data for the Dallas & Fort Worth area only 1% of our population was of Ashkenazi Jewish ancestry. Based on the diversity and size of our mammogram populations, we predicted there would be 47 and 85 mutation carriers in the insured and underserved populations, respectively.

### 2.4 Predictive Model of Cancer Reduction in HBOC Patients

Using 186,537 female patients between the ages of 18 and 85 seen at Parkland and JPS hospitals in 2009 as the simulation population, we developed models for breast and ovarian cancer that would predict the cancers in patients with mutations in the *BRCA* genes based on the expected age distribution in this population. This simulation population was used to estimate how many cases of breast and ovarian cancer we expected to find in mutation carriers versus what we actually found given the age distribution of our patients. Predictions of cancer incidence in patients who had not had preventative surgeries were calculated from the cumulative cancer incidence for the *BRCA1* and *BRCA2* mutation carriers based on reported per year penetrance data (Chen and Parmigiani, 2007). We defined the “at risk” women as those between ages 35 and 85 who had a *BRCA* mutation but did not have breast or ovarian cancer at the start of the follow up period (this was calculated by subtracting out the prevalence of breast/ovarian cancers at each age interval). These at risk women would then be, in simulation, followed up to age 85. The number of breast/ovarian cancers that would be diagnosed each year at each age interval was calculated by continually resetting the number “at risk” after each year, and by subtracting out the number who developed breast/ovarian cancer in the prior year. For the calculation of breast cancer prevention, we also subtracted 50% of the breast cancers in the prior year if bilateral prophylactic oophorectomy was completed in the patient before age 50 (Rebbeck et al., 2009).

**Fig. 2:**
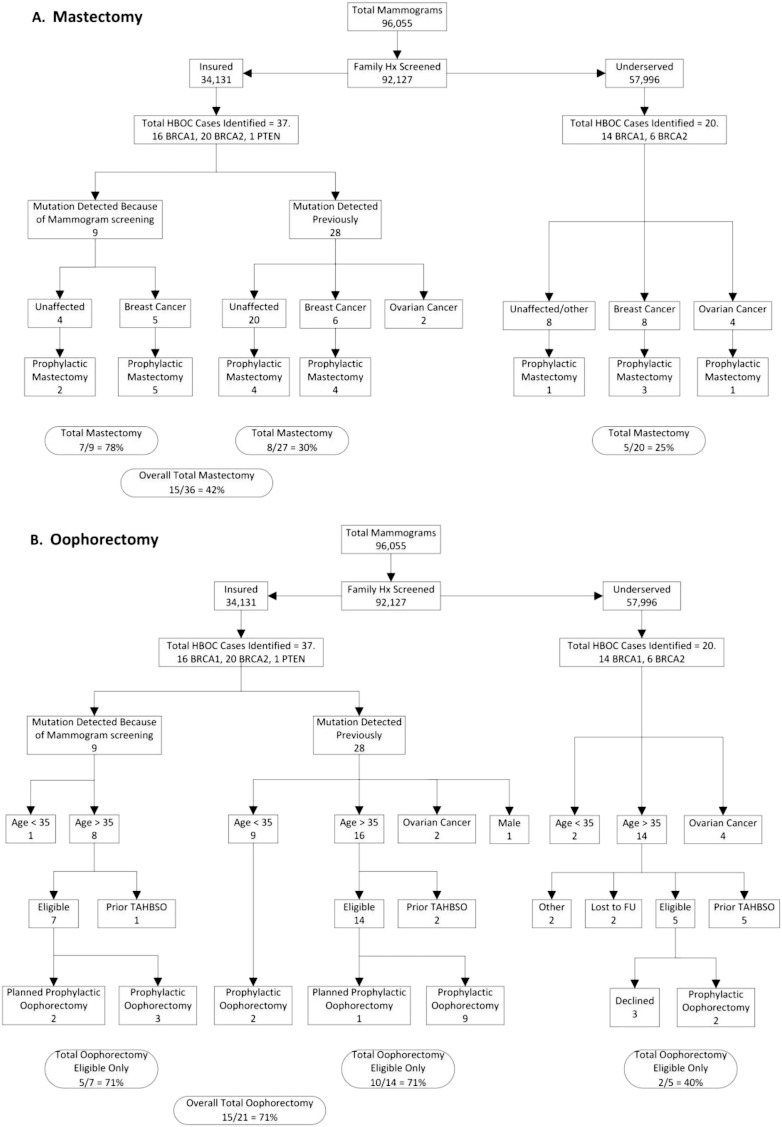
Rates of prophylactic surgeries in HBOC patients. Mutation carriers in the insured population (n = 37) and in the underserved population (n = 20) were evaluated for history of A) prophylactic mastectomy or B) prophylactic oophorectomies. The one male insured patient was excluded from the calculations of prophylactic mastectomy rates and NCCN compliance since guidelines do not exist.

Applying our mutation detection (Fig. 1) and uptake of prophylactic surgery rates (Fig. 2), we calculated the predicted reduction of breast and ovarian cancer incidence over 30 years. In our calculations, bilateral prophylactic mastectomy was assumed to reduce the risk of breast cancer by 95% and bilateral oophorectomy (prior to age 50) was assumed to reduce the risk of breast cancer by 50% and ovarian cancer by 95% (Rebbeck, TR, et al., 2004, Rebbeck, TR, et al., 2002, Friebel, TM, et al., 2007). A software calculator was written using R language specifically for this use (see Supplementary data).

## 3 Results

### 3.1 Mammography Screening for HBOC

Of 96,055 mammogram patients, 34,131 were from the reference population from the private hospital (Fig. 1A; “insured”) and 61,924 were from the county hospitals (Fig. 1B; “underserved”). The participation rate in completing the screening tool was 95.9% (92,127 cases reviewed). Five percent were identified, based on the RST screen, as high-risk in each population. Fifty-one percent of high-risk patients in the underserved population accepted a referral to genetic counseling, but only half of those kept their appointment (Fig. 1B). Twelve percent of the insured patients had already been seen by our genetics group, compared to none in the underserved population. Thirteen percent of insured patients never seen by genetics were referred to genetic counseling. Overall, 25% of identified high-risk insured patients were seen for genetic testing and 85% of those referred kept the scheduled appointment (Fig. 1A).

### 3.2 Population Diversity

In the underserved population, 58.4% were Caucasian, 37.3% African American, 3% Asian, and < 2% other. Twenty-seven percent of the underserved reported Hispanic ancestry. In the insured mammogram screening reference population, 66.5% were Caucasian, 18.5% African American, 13% other, and 2% Asian and Indian. Hispanic ancestry was reported in 10.8% of insured patients.

### 3.3 Genetic Analysis and HBOC Mutation Detection Rate

Seventy-six percent (446/589) of the underserved patients who were referred due to their family history of cancer underwent BRCA gene testing. The remaining patients either declined testing or testing was not indicated after further consultation. Overall, 4.5% of referred patients (20/446) were found to have a germline mutation.

We identified only 23.5% (20/85) of predicted *BRCA* mutation carriers in the underserved cohort (Fig. 1B). All of these 20 patients were newly diagnosed with HBOC syndrome as a result of the mammography screen and 12 had either a prior (n = 7) or concomitant (n = 5) diagnosis of cancer (60% (12/20)). In the insured reference population, we identified 36 of the 47 (76.6% of expected) predicted *BRCA* mutation carriers (Fig. 1A). Of the 9 newly identified HBOC syndrome patients, 5 had either a prior (n = 3) or concomitant (n = 2) diagnosis of breast cancer (55% (5/9)).

### 3.4 Surveillance Imaging

We evaluated all female *BRCA* mutation carriers' compliance to the 2012 NCCN guidelines for surveillance. Clinical data were obtained from a chart review through June 2015, with a range of follow-up from 24 to 44 months. Thirty-nine percent of the underserved HBOC patients met the NCCN management recommendations for surveillance. If a patient was under active treatment for cancer or if they had stage IV disease and a mammogram or MRI appointment was missed, we did not count it as noncompliance with the NCCN guidelines since this “screening” was not a priority. Twenty-two percent of the underserved patients were not offered surveillance imaging (MRI/mammography) because of metastatic cancer. In the patients who did not meet NCCN guidelines, 32% of patients had no record of an annual mammogram or MRIand 6% of patients declined all follow-up. In the insured reference population with HBOC syndrome that were followed at our institution, the patient compliance to the NCCN management guidelines was 72%.

### 3.5 Cancer Stage of Mutation Carriers

Of the 12 underserved patients with HBOC syndrome with cancer, the average age was 52 and 83% were minorities. Of the four patients with ovarian cancer, all had a stage III disease. Of the 8 breast cancer patients, one was at stage I, one was at stage II, four were at stage III, one was stage IV and one patient's cancer stage was unknown. Overall, 82% of uninsured patients with known staging had an advanced cancer (stage III or IV).

Thirteen of the insured patients with HBOC syndrome had cancer, two with ovarian and eleven with breast cancer, prior to or at mammogram screening. The average age was 51.9 and 23% were minorities. Of the two cases of ovarian cancer, both were stage III. Of the insured breast cancer patients, three were stage 0, four were stage I, three were stage II and in one case, stage was not available. There were no insured breast cancer patients with advanced stage cancer (stage III or IV). Twenty-two percent of insured patients with known staging had an advanced cancer.

### 3.6 Prophylactic Surgery

We next assessed whether patients had prophylactic mastectomies or oophorectomies (Fig. 2). Of the insured reference patients in whom a genetic mutation was detected prior to the mammogram screening program, 30% elected to have prophylactic mastectomies. Seventy-eight percent of the mutation carriers identified as a result of the mammogram screening program elected prophylactic mastectomy, with the overall rate in the insured population of 40.5%. Within the public hospital setting, 37.5% of the cancer patients elected prophylactic contralateral mastectomy and 12.5% of the unaffected patients elected prophylactic bilateral mastectomies (25% overall) (Fig. 2A). In regard to prophylactic oophorectomy, 71% of the insured *eligible* (i.e. those patients over the age of 35 with at least one intact ovary) mutation carriers newly identified by the RST screen (N = 5/7) elected to have surgery and 71% of the previously identified mutation carriers elected the same (N = 10/14). For the uninsured population, 40% of the identified *eligible* HBOC patients (N = 2/5) elected to have prophylactic oophorectomies (Fig. 2B). All patients with a prior history of oophorectomy for unrelated medical reasons were excluded from this analysis.

**Fig. 3:**
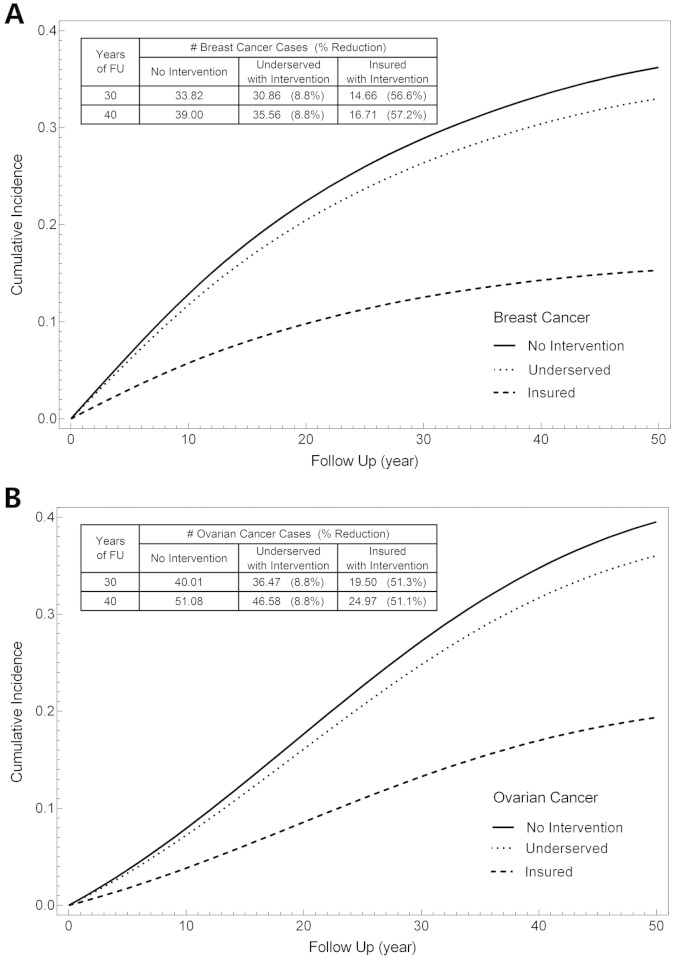
Modeling risk reduction for HBOC. Simulation models for cancer prevention in the different populations. Solid line represents no intervention as a result of genetic testing, dotted line represents the underserved population and dashed line represents the insured population. A) HBOC patients were reviewed for whether or not they underwent prophylactic mastectomy and then this frequency was used to model breast cancer risk reduction over 30 or 40 years. B) HBOC patients were reviewed for whether or not they underwent prophylactic oophorectomies and then this frequency was used to model ovarian cancer risk reduction over 30 or 40 years.

Based on the uptake of prophylactic surgeries in *BRCA* mutation carriers, we predicted an insignificant 8.8% reduction in breast and ovarian cancer in the underserved population. In contrast, in the insured reference population of identified *BRCA* mutation carriers, cancer risk reduction was significantly greater at 56.6% for breast cancer (Fig. 3A; p < 0.0075, chi-square, 30 year follow up) and 51.3% for ovarian cancer (Fig. 3B; p < 0.0117, chi-square, 30 year follow up) if the proposed mutation detection rate, with all of the caveats associated with this calculation, was truly 76.6%.

## 4 Discussion

The aim of our study was to determine if screening for a family history of cancer in underserved mammography populations would identify patients at high risk for HBOC syndrome that would then lead to genetic testing and increased cancer prevention. We found low levels of genetic screening and adherence to management guidelines in the underserved HBOC syndrome patients. The number of unaffected patients in the underserved population identified as *BRCA1*/*2* mutation carriers was lower than our reference insured population and those who were identified had fewer preventative surgeries. The data were used to model cancer prevention frequency and a significant decrease in cancer incidence was only found in the insured reference population.

Our model calculates the reduction of cancer incidence in HBOC patients by taking the population's age distribution, mutation detection rate and frequency of prophylactic surgery into account. From these data, our model predicted only an 8.8% reduction in the both the breast and ovarian cancer rate over 30 years in the underserved. Although we did not have a true control population of insured patients managed by a single institution, the mutation detection rate and the rate of prophylactic surgeries were higher in this population. Using the data from this “reference” population we illustrate how the model can predict that increasing mutation detection frequency and prophylactic surgery rates can be used as benchmarks for increasing cancer prevention in patients with HBOC syndrome.

Although this model only addresses the reduction in cancer incidence over time based on the prophylactic surgery and mutation detection rates, patient compliance to other NCCN management recommendations for breast cancer surveillance can contribute to the early detection of breast cancers in patients with HBOC syndrome. The compliance with NCCN HBOC syndrome management guidelines in the underserved population studied here was only 39%. Improved genetic navigation and further research to understand and quantify patient barriers to care may increase the NCCN compliance rates in these patients. General barriers for health care in the underserved such as financial constraints, transportation challenges, limited education and inability to take time off work are obvious candidates. The reduced compliance of the underserved population with NCCN surveillance guidelines mirrors the reduced uptake of genetic referrals and low completion rates of prophylactic surgeries.

For the underserved mammography patients, but not the insured patients, genetic navigators were available to contact and track patients identified as high-risk. The underserved patients are likely to benefit more from services of a genetic navigator because they are often more difficult to reach and less informed than the insured population. On a more practical note, funds for genetic navigators in this study were not available for the insured reference population. The lack of navigation for the insured may be a contributing factor to the surprisingly low frequency of referred insured patients (Fig. 1A). Further, although chart documentation of genetic counseling was absent in 70% of the high-risk insured patients recommended for genetic counseling, this does not mean they did not have genetic analysis at an outside site. In fact, at least 20% of insured mammogram patients are referred to UT Southwestern from outside providers, making it unknown whether the family history of cancer led them to receive genetic analysis at different institutions. However, if these analyses were done on the outside, the mutation rate in the insured population would only increase and make the differences between the insured and the underserved cohorts greater.

Without knowing the outcomes of the non-referred patients, we limited comparisons of the insured and underserved patients to those who had documented genetic analyses at our institution. Although this is a study limitation, we were comfortable in our use of these data in our model because when compared to other reports of rates of preventative measures taken, they are similar to our reference population (Skytte, AB, et al., 2010, Singh, K, et al., 2013, Schwartz, MD, et al., 2012). The published rates from smaller populations are also higher than the rates we observed in our underserved population emphasizing the greater need for improvement in the underserved.

There was one area of cancer prevention where the underserved fared better than the insured reference population and that was the rate of documented referral of patients for genetic evaluation. This may have been because the patient navigator was available for the underserved patients but not for the insured patients. Certainly, if a navigator had not been used for the underserved, we would have identified even fewer *BRCA* mutation carriers.

We were surprised by the large fraction of insured patients who did not have documented genetic analysis despite their positive family history of cancer. Possible explanations for this low frequency include: 1) these patients were counseled and tested at another hospital; 2) the primary doctor and patient revised the original family history so that genetics referral was no longer warranted; or 3) the patients should have been referred and were not. For the underserved populations, patients were easier to follow as our safety net hospitals systems (Parkland Memorial, and JPS) are the only places these patients receive affordable health care. Genetic testing for cancer predisposition is not a simple decision and as shown here, efforts to navigate all of our patients (not just the underserved) and educate both patients and their physicians, to improve cancer prevention, is needed.

Another potential limitation of our computer model is that we included prophylactic oophorectomy as a factor that predicts breast cancer reduction by 50% in *BRCA1*/*2* mutation carriers (Rebbeck et al., 2009). A recent report cited potential bias in many of the past studies that predicted oophorectomy reduced breast cancer risk (Heemskerk-Gerritsen et al., 2015). At this time, more long term data are needed to understand the impact of oophorectomy on breast cancer incidence in *BRCA1*/*2* mutation carriers.

Although screening for a high genetic risk for cancer saves not only the patient's life but also their family members' lives, it is likely many unaffected individuals and their physicians are unaware of the benefits of knowing their genetic risks. For example, we were surprised at the number of newly identified HBOC patients who had a prior history of breast or ovarian cancer but had not had a genetic analysis (35% (7/20) of underserved and 33% (3/9) of insured patients). Screening of primary care and mammography populations will continue to identify affected patients who had not previously been offered genetic screening; this emphasizes that screening for *BRCA1*/*2* mutation carriers must be multifaceted and not just occur in the oncology clinic.

Decreasing the cancer incidence in HBOC patients or other hereditary cancer syndromes can only be achieved by evaluation of the entire screening progress. Offering women *BRCA1*/*2* gene testing is not enough to decrease cancer risk on a population basis. There are two main areas that contribute to the decreased cancer prevention in the underserved HBOC patients. First, we found that only a quarter of the underserved patients came in for genetic analysis. Further research is needed to determine reasons patients decline genetic analysis and potential testing. Second, if they do come in, a need for an increased acceptance of prophylactic oophorectomies and mastectomies will increase the prevention of ovarian and breast cancer incidence.

In sum, even though genetic testing to identify *BRCA1*/*2* mutation carriers has been integral to oncology practices for almost 20 years, little data have been reported on the outcomes of these genetic screening programs in large diverse primarily unaffected populations. Although we identified only twenty underserved patients with HBOC syndrome through mammography screening of 61,924 patients, this is the largest underserved population at risk that has been identified at mammography and followed for the preventative measures taken. By isolating and quantifying key steps to cancer risk reduction, we have established a preliminary baseline quality measure to calculate where we stand and to effectively guide us as we move to 100% compliance with evidenced-based guidelines.

## Author Contributions

LSR and TSR collected, organized and interpreted data and wrote the manuscript. AH, SPM and MP collected, organized and interpreted data and edited the manuscript. PR and SP collected data. XJX, JY and DE interpreted data and edited the manuscript.
