# Supplementary material for: Prediction of Cancer Prevention: From Mammogram Screening to Identification of BRCA1/2 Mutation Carriers in Underserved Populations
Source: eBioMedicine. 2015 Oct 21;2(11):1827–33. doi: 10.1016/j.ebiom.2015.10.022 (PMC4740331; doi:10.1016/j.ebiom.2015.10.022)
Supplement: Supplementary file 1 — Supplementary material [file mmc1.zip › Explanation of Input data as it relates to the calculators.docx]

The data input files have the same names as in the R code calculators.  Simply place the files in the same folder as the R code and run the R code. Explanation of the four different input files:

1. Population data: both pieces of R code share the same population data as in this file.
2. Penetrance data: this file contains penetrance data of BRCA1 and BRCA2 mutations for both breast cancer and ovarian cancer in different columns and each piece of the R code will automatically pick up the correct column to use.
3. OBPrivate file: data in this file provides the effect of oophorectomy on breast cancer incidence for subjects in the insured group.
4. OBPublic file: data in this file provide the effect of oophorectomy on breast cancer incidence for subjects in the underserved group.
