# Supplementary material for: Prediction of Cancer Prevention: From Mammogram Screening to Identification of BRCA1/2 Mutation Carriers in Underserved Populations
Source: eBioMedicine. 2015 Oct 21;2(11):1827–33. doi: 10.1016/j.ebiom.2015.10.022 (PMC4740331; doi:10.1016/j.ebiom.2015.10.022)
Supplement: Supplementary file 1 — Supplementary material [file mmc1.zip › Ovca Prevention calculator.pdf]

```
Sub1 <- function()
{
Z2_data1 <- read.csv("PenetranceData.csv",header=T)
Z2_data2 <- matrix(NA,nr=110,nc=4)
for(i in 1:110)
  for(j in 1:4)
    Z2_data2[i,j] <- sum(Z2_data1[1:i,j+1])
Z2_out <- cbind(Z2_data1,Z2_data2)
return( list(A=Z2_out))
}




Sub2 <- function(Z2_out)
{
Z1_data1<-read.csv("PopulationData_new.csv",header=T)
Z1_out<-matrix(NA,nr=110,nc=20)
Z1_out[,1]  <- Z1_data1[,1]
Z1_out[,2]  <- Z1_data1[,2]
Z1_out[,3]  <- Z1_data1[,3]
Z1_out[,4]  <- Z1_data1[,2]+Z1_data1[,3]
Z1_out[,5]  <- Z1_out[,4]*0.01
Z1_out[,6]  <- Z1_out[,4]-Z1_out[,5]
Z1_out[,7]  <- Z1_out[,5]*0.0061
Z1_out[,8]  <- Z1_out[,5]*0.0068
Z1_out[,9]  <- Z1_out[,6]*0.000583
Z1_out[,10] <- Z1_out[,6]*0.00068
Z1_out[,11] <- Z1_out[,7]+Z1_out[,9]
Z1_out[,12] <- Z1_out[,8]+Z1_out[,10]
Z1_out[,13] <- Z1_out[,11]*Z2_out[,6]
Z1_out[,14] <- Z1_out[,12]*Z2_out[,7]
Z1_out[,15] <- Z1_out[,11]*Z2_out[,8]
Z1_out[,16] <- Z1_out[,12]*Z2_out[,9]
Z1_out[,17] <- Z1_out[,11]-Z1_out[,13]
Z1_out[,18] <- Z1_out[,12]-Z1_out[,14]
Z1_out[,19] <- Z1_out[,11]-Z1_out[,15]
Z1_out[,20] <- Z1_out[,12]-Z1_out[,16]
Z1_outRow <- matrix(NA,nr=1,nc=20)
for(i in 4:20) Z1_outRow[i] <- sum(Z1_out[18:85,i])
return( list(A=Z1_out,C=Z1_outRow))
}




Common <- function(Z1_out,Z2_out,mutIDrate,UptakeRate,NNN)
{

ZZ_b <- paste("FU",c(1:50),sep="")
ZZ_out1 <- matrix(NA,nr=110,nc=51)
colnames(ZZ_out1) <- c("Age",ZZ_b)
ZZ_out1[,1] <- c(1:110)

ZZ_out2 <- matrix(NA,nr=110,nc=52)
ZZ_out2[,1] <- c(1:110)
if (is.element(NNN,c(3,4,5,6)))
  {
  if (NNN==4)  ZZ_c1 <- Z1_out[,20] else  ZZ_c1 <- Z1_out[,19]
  for(i in c(1:17,86:110)) ZZ_c1[i] <- NA
  ZZ_out2[,2] <- ZZ_c1
  }

ZZ_out3 <- matrix(NA,nr=110,nc=2)
ZZ_out3[,1] <- c(1:110)
if (is.element(NNN,c(3,5,6,7)))  ZZ_d1 <- Z2_out[,4]
if (NNN == 8)  ZZ_d1 <- Z2_out[,4]*0.05
if (is.element(NNN,c(4,9)))  ZZ_d1 <- Z2_out[,5]
if (NNN == 10)  ZZ_d1 <- Z2_out[,5]*0.05
if (is.element(NNN,c(3,4,5,6)))  for(i in 1:17) ZZ_d1[i] <- NA
if (is.element(NNN,c(7,8,9,10)))  for(i in 1:34) ZZ_d1[i] <- NA
if (NNN==6)  for (i in 35:110) ZZ_d1[i] <- ZZ_d1[i]*0.05
ZZ_out3[,2] <- ZZ_d1

if (is.element(NNN,c(5,6,7,8,9,10)))
```

```
  {
  ZZ_out4 <- matrix(NA,nr=110,nc=3)
  ZZ_out4[,1] <- c(1:110)
  for(i in 35:85)
    {
    if (is.element(NNN,c(5,6,7,8)))  ZZ_out4[i,2] <- Z1_out[i,19]
    if (is.element(NNN,c(9,10)))  ZZ_out4[i,2] <- Z1_out[i,20]
    if (is.element(NNN,c(5,7,9)))
      ZZ_out4[i,3] <- ZZ_out4[i,2] - ZZ_out4[i,2]*mutIDrate*UptakeRate
    if (is.element(NNN,c(6,8,10)))
      ZZ_out4[i,3] <- ZZ_out4[i,2]*mutIDrate*UptakeRate
    ZZ_out2[i,2] <- ZZ_out4[i,3]
    }
  }

if (is.element(NNN,c(3,4,5,6))) msa <- 18
if (is.element(NNN,c(7,8,9,10))) msa <- 35
for(rr in msa:85) ZZ_out1[rr,2] <- ZZ_out2[rr,2]*ZZ_out3[rr,2]
for(cc in 3:51)
  {
  for(rr in msa:85)
    {
    ZZ_out2[rr,cc] <- ZZ_out2[rr,cc-1]-ZZ_out1[rr,cc-1]
    if (rr+cc<=112) ZZ_out1[rr,cc] <- ZZ_out2[rr,cc]*ZZ_out3[rr+cc-2,2]
    else ZZ_out1[rr,cc] <- 0
    }
  }
for(rr in msa:85) ZZ_out2[rr,52] <- ZZ_out2[rr,51]-ZZ_out1[rr,51]

ZZ_outRow <- matrix(NA,nr=1,nc=51)
colnames(ZZ_outRow) <- c("Age",ZZ_b)
if (is.element(NNN,c(3,4,5)))
  for(i in 2:51) ZZ_outRow[i] <- sum(ZZ_out1[18:85,i])
if (is.element(NNN,c(6,7,8,9,10)))
  for(i in 2:51) ZZ_outRow[i] <- sum(ZZ_out1[35:85,i])

ZZ_outRowPartial <- matrix(NA,nr=1,nc=51)
colnames(ZZ_outRowPartial) <- c("Age",ZZ_b)
for(i in 2:51) ZZ_outRowPartial[i] <- sum(ZZ_out1[35:85,i])

return(list(A=ZZ_out1,B=ZZ_out2,C=ZZ_outRow,D=ZZ_outRowPartial))
}




Sub11 <- function()
{
out02 <- Sub1()
out01 <- Sub2(Z2_out=out02$A)
out03 <- Common(Z1_out=out01$A,Z2_out=out02$A,mutIDrate,UptakeRate,NNN=3)
out04 <- Common(Z1_out=out01$A,Z2_out=out02$A,mutIDrate,UptakeRate,NNN=4)


Z11_a1 <- matrix(NA,nr=50,nc=19)
Z11_out1 <- as.data.frame(Z11_a1)
Z11_out1$V1[1:8] <- c("BRCA1 mut","BRCA2 mut","prevBRCA1Br","PrevBRCA2Br",
                      "Total prev Br","BRCA1AtRiskBr","BRCA2AtRiskBr",
                      "Total At Risk")
Z11_out1$V2[1:4] <- c(round(out01$C[11],digits=0), round(out01$C[12],digits=0),
                      round(out01$C[15],digits=0), round(out01$C[16],digits=0))
Z11_out1$V2[5:7] <- c(Z11_out1$V2[3]+Z11_out1$V2[4], Z11_out1$V2[1]-Z11_out1$V2[3],
                      Z11_out1$V2[2]-Z11_out1$V2[4])
Z11_out1$V2[8] <- Z11_out1$V2[1]+Z11_out1$V2[2]-Z11_out1$V2[5]
Z11_out1[1:4,3:12] <- ''
Z11_out1[5,3:12] <- c('>=35','5%','10%','15%','20%','25%','30%','35%','40%','50%')
Z11_out1[6,3] <- round(sum(out01$A[35:85,19]),digits=0)
Z11_out1[7,3] <- round(sum(out01$A[35:85,20]),digits=0)
Z11_out1[8,3] <- as.numeric(Z11_out1[6,3])+as.numeric(Z11_out1[7,3])
for(i in 4:12)
  {
  Z11_out1[6,i] <- as.numeric(Z11_out1[6,3]) * (1/100) *
                   as.numeric(unlist(strsplit(Z11_out1[5,i],"%")))
  Z11_out1[7,i] <- as.numeric(Z11_out1[7,3]) * (1/100) *
                   as.numeric(unlist(strsplit(Z11_out1[5,i],"%")))
  Z11_out1[8,i] <- as.numeric(Z11_out1[6,i]) + as.numeric(Z11_out1[7,i])
  }
```

```
Z11_out2A <- matrix(NA,nr=50,nc=4)
Z11_out2A[,1] <- c(1:50)
Z11_out2A[,2] <- out03$C[2:51]
for(i in 1:50)
  {
  if (i==1) Z11_out2A[1,3] <- Z11_out2A[1,2]
  else Z11_out2A[i,3] <- Z11_out2A[i,2]+Z11_out2A[i-1,3]
  Z11_out2A[i,4] <- Z11_out2A[i,3]/Z11_out1[6,2]
  }
colnames(Z11_out2A) <- c("FU","N Br","Cumm Br","Cumm %")


Z11_out2B <- matrix(NA,nr=50,nc=4)
Z11_out2B[,1] <- c(1:50)
Z11_out2B[,2] <- out04$C[2:51]
for(i in 1:50)
  {
  if (i==1) Z11_out2B[1,3] <- Z11_out2B[1,2]
  else Z11_out2B[i,3] <- Z11_out2B[i,2]+Z11_out2B[i-1,3]
  Z11_out2B[i,4] <- Z11_out2B[i,3]/Z11_out1[7,2]
  }
colnames(Z11_out2B) <- c("FU","N Br","Cumm Br","Cumm %")


Z11_out2C <- matrix(NA,nr=50,nc=4)
Z11_out2C[,1] <- c(1:50)
for(i in 1:50)
  {
  Z11_out2C[i,2] <- Z11_out2A[i,2]+Z11_out2B[i,2]
  if (i==1) Z11_out2C[1,3] <- Z11_out2C[1,2]
  else Z11_out2C[i,3] <- Z11_out2C[i,2]+Z11_out2C[i-1,3]
  Z11_out2C[i,4] <- Z11_out2C[i,3]/Z11_out1[8,2]
  }
colnames(Z11_out2C) <- c("FU","N Br","Cumm Br","Cumm %")


Z11_out3A <- matrix(NA,nr=50,nc=4)
Z11_out3A[,1] <- c(1:50)
for(i in 1:50)
  {
  Z11_out3A[i,2] <- out03$D[1,i+1]
  if (i==1) Z11_out3A[i,3] <- Z11_out3A[i,2]
  else Z11_out3A[i,3] <- Z11_out3A[i-1,3]+Z11_out3A[i,2]
  Z11_out3A[i,4] <- Z11_out3A[i,3]/as.numeric(Z11_out1[6,3])
  }


Z11_out3B <- matrix(NA,nr=50,nc=4)
Z11_out3B[,1] <- c(1:50)
for(i in 1:50)
  {
  Z11_out3B[i,2] <- out04$D[1,i+1]
  if (i==1) Z11_out3B[i,3] <- Z11_out3B[i,2]
  else Z11_out3B[i,3] <- Z11_out3B[i-1,3]+Z11_out3B[i,2]
  Z11_out3B[i,4] <- Z11_out3B[i,3]/as.numeric(Z11_out1[7,3])
  }


Z11_out3C <- matrix(NA,nr=50,nc=4)
Z11_out3C[,1] <- c(1:50)
for(i in 1:50)
  {
  Z11_out3C[i,2] <- Z11_out3A[i,2]+Z11_out3B[i,2]
  if (i==1) Z11_out3C[i,3] <- Z11_out3C[i,2]
  else Z11_out3C[i,3] <- Z11_out3C[i-1,3]+Z11_out3C[i,2]
  Z11_out3C[i,4] <- Z11_out3C[i,3]/as.numeric(Z11_out1[8,3])
  }


Z11_out1[,13] <- Z11_out2C[,1]
Z11_out1[,14] <- Z11_out2C[,4]
Z11_out1[,15] <- Z11_out3C[,4]
return( Z11_out1)
}



MainSub <- function(mutIDrate,UptakeRate)
```

```
{
out02 <- Sub1()
out01 <- Sub2(Z2_out=out02$A)
for(NNN in 7:10)
  assign( paste("out",NNN,sep=""),
          Common(Z1_out=out01$A,Z2_out=out02$A,mutIDrate,UptakeRate,NNN) )
sumRow <- out7$C + out8$C + out9$C + out10$C
return(sumRow)
}




##############################    PRIVATE    ##########################
setwd("C:/Users/Desktop/ZZZZ")
UptakeRate <- 0.71
mutIDrate <- 0.767


Summary <- Sub11()
Summary <- Summary[,c(-17,-18,-19)]
MainSubout <- MainSub(mutIDrate,UptakeRate)
aa <- matrix(NA,nr=50,nc=3)
for(i in 1:50)
  {
  aa[i,1] <- MainSubout[i+1]
  if(i==1) aa[i,2] <- aa[i,1]
  else aa[i,2] <- aa[i-1,2]+aa[i,1]
  aa[i,3] <- aa[i,2]/as.numeric(Summary[8,3])
  }
Summary[,16] <- aa[,3]
bb<-matrix(c(rep(NA,12),rep(0,4)),nr=1,nc=16)
Summary1 <- rbind(bb,Summary)
write.csv(Summary1,"outNewOvaryPrivate.csv",row.names=F,na="")




##############################    PUBLIC    ##########################
setwd("C:/Users/Desktop/ZZZZ")
UptakeRate <- 0.4
mutIDrate <- 0.235


Summary <- Sub11()
Summary <- Summary[,c(-17,-18,-19)]
MainSubout <- MainSub(mutIDrate,UptakeRate)
aa <- matrix(NA,nr=50,nc=3)
for(i in 1:50)
  {
  aa[i,1] <- MainSubout[i+1]
  if(i==1) aa[i,2] <- aa[i,1]
  else aa[i,2] <- aa[i-1,2]+aa[i,1]
  aa[i,3] <- aa[i,2]/as.numeric(Summary[8,3])
  }
Summary[,16] <- aa[,3]
bb<-matrix(c(rep(NA,12),rep(0,4)),nr=1,nc=16)
Summary1 <- rbind(bb,Summary)
write.csv(Summary1,"OutNewOvaryPublic.csv",row.names=F,na="")
```
